# Asthma exacerbations and traffic: examining relationships using link-based traffic metrics and a comprehensive patient database

**DOI:** 10.1186/s12940-016-0184-2

**Published:** 2016-11-03

**Authors:** Paula Lindgren, Jean Johnson, Allan Williams, Barbara Yawn, Gregory C. Pratt

**Affiliations:** 1 Minnesota Department of Health, Chronic Disease and Environmental Epidemiology, PO Box 64882, St. Paul, MN 55164-0882 USA; 2 Olmsted Medical Center, 210 Ninth Street SE, Rochester, MN 55904 USA; 3 Environmental Outcomes Division, Minnesota Pollution Control Agency, 520 Lafayette Road, St. Paul, MN 55155 USA; 4 Division of Environmental Health, University of Minnesota, School of Public Health, Mayo Mail Code 197, 420 Delaware St. S.E., Minneapolis, MN 55455-0381 USA

**Keywords:** Asthma, Traffic, Epidemiology, Longitudinal, Cross section

## Abstract

**Background:**

The Rochester Epidemiology Project (REP) is a unique community-based medical record data linkage system that provides individual patient address, diagnosis and visit information for all hospitalizations, as well as emergency department, urgent care and outpatient clinic visits for asthma. Proximity to traffic is known to be associated with asthma exacerbations and severity. Our null hypothesis was that there is no association between residential proximity to traffic and asthma exacerbations over eleven years of REP data.

**Methods:**

Spatial coordinates of the homes of 19,915 individuals diagnosed with asthma were extracted from the REP database. Three metrics of traffic exposure at residences were calculated from link-based traffic count data. We used exploratory statistics as well as logistic and Poisson regression to examine associations between three traffic metrics at the home address and asthma exacerbations.

**Results:**

Asthma exacerbations increased as traffic levels near the home increased. Proximity to traffic was a significant predictor of asthma exacerbations in logistic and Poisson regressions controlling for age, gender and block group poverty.

**Conclusions:**

Over eleven years in a comprehensive county-wide data set of asthma patients, and after controlling for demographic effects, we found evidence that living in proximity to traffic increased the risk of asthma exacerbations.

## Background

There is a growing body of evidence linking exposure to traffic-related air pollution with asthma prevalence [1–8] and morbidity [9–14]. These findings are superimposed on pre-existing vulnerabilities related to socio-economic status [9, 15–23]. Traffic is comprised of a complex mixture of air pollutants, and other stressors such as noise and elevated levels of activity, that are highly variable in time and space. It is likely that multiple, interacting components of traffic are involved in specific adverse health effects such as asthma, although etiologies are only partially understood. Traffic emissions of ultrafine and nanoparticles are among the more short-lived components in space and time, and recent work [2, 3, 24–27] suggests that they may be particularly harmful. Since ultrafine and nonparticles disperse and coagulate quickly downwind from sources, studies to assess exposure and health effects should be focused near sources of emissions. To date there are relatively few measurements of the occurrence and composition of these smallest sized particles, and there is no consensus on the best metric for relating them to adverse health impacts. Studies that look at the relationship between a single traffic-related air pollutant and asthma may not capture the cumulative effects of traffic on disease outcome. We chose to focus on exposure to traffic using three levels of spatial resolution. This approach provides some limited information on the spatial scale of the stressor causing asthma exacerbations.

Asthma prevalence in the US stands at 8.0 % among adults and 9.3 % among children [28]. Minnesota rates differ slightly from national rates in that approximately 7.7 % of adults and 7.0 % of children currently have asthma [29]. This study was part of a larger project to develop and evaluate outcome-based indicators for monitoring the impacts of local, regional and national particulate matter emission-reduction strategies on ambient PM exposures, and on population health in the seven county Minneapolis-St. Paul metropolitan area (MSP) and Olmsted County in Minnesota. In this sub-study of Olmsted County, the Rochester Epidemiology Project (REP) offered a unique patient data set and to explore the development and application of spatially refined indicators of traffic-related exposure.

Located about 150 km southeast of MSP, Rochester is the third largest city in the State of Minnesota and the county seat of Olmsted County. The population of Olmsted County was 141,244 (83 % urban, with 106,769 in the city of Rochester) in the 2010 census over a land area of 1691 km^2^ resulting in an average density of 83.5 persons per km^2^. There are 111 block groups in Olmsted County ranging in size from 0.19 to 219 km^2^ and in population density from 3 to 45,000 persons per km^2^. According to the Minnesota Geospatial Information Office [30] county land use is categorized as cultivated (55 %), pasture (24 %), forest/brush land (11 %), and urban/rural developed (9 %). Annual average daily traffic counts (AADT) reach 81,000 on the busiest roadway segments in the city, compared with 210,000 on the busiest roadway segments in the Minneapolis-St. Paul metropolitan area [31].

The REP is a community-based medical record data linkage system covering virtually all individuals in the county receiving health care. It provides patient address, diagnosis and visit information for all hospitalizations, as well as emergency department, urgent care and outpatient clinic visits for asthma. The individual level data allowed us to utilize address-specific residence locations for developing three metrics of traffic exposure to compare with the frequency of asthma exacerbations in each case.

The objective of this study was to evaluate whether asthma exacerbations in children and adults were affected by traffic exposure determined at the residence while controlling for individual demographics and block group poverty level. We also sought to develop and demonstrate the utility of a spatially-refined indicator of traffic related exposure. We did not look at or control for the effects of individual air pollutants or combinations of pollutants, the exposure metric was traffic itself.

## Methods

### Study population

The REP is a medical record linkage system of virtually all Olmsted County, Minnesota residents and extends into adjacent counties (Kurland et al. [32]; Rocca et al. [33]; St Sauver et al. [34]). Data from all health care visits to any health care facility (office, hospital, outpatient clinic, urgent care or emergency department) are collected within the REP and linked across healthcare facilities for each individual Olmsted County resident who has provided research authorization [35–37]. This allows for the collection of community population-based data on all health services used, since over 98 % of all health services provided to Olmsted County residents occurs within the county and over 95 % of all residents have not denied research authorization [37]. Over 96 % of the population had some type of insurance at all times covered by the study period.

Movement into and out of the study area varied by age group [34] with highest rates in the preschool ages when parents often finish training or education and among those in late teens and early 20s who move away from home (both groups ~13 % per year). The rates of leaving the area are around 4 % in all other age groups. Gaps in insurance status are tracked via billing data. Residents’ mobility within the county is limited by the available housing within price ranges. Most low income housing is concentrated within specific areas of the city which are adjacent to higher traffic areas. The limitation of having a single address for the entire time period is likely mitigated by the concentrated area in which individuals are likely to move, especially those in areas with high traffic patterns.

The outcome of interest for this study was exacerbation of asthma. Individuals were selected from the REP data linkage system who had at least two visits with asthma as the first diagnosis (ICD-9-CM code 493) within any 18 month period during 2000–2010 (*n* = 19,915 individuals). We used the two asthma codes within 18 months to ensure that the codes were not in error or part of differential diagnosis, as may happen when a single asthma-related code is found. Asthma exacerbations were defined as one of three types of events: 1) an inpatient hospitalization for asthma, 2) an emergency department (ED) visit for asthma, or 3) three or more outpatient visits for asthma within a 2-week time period [38]. Asthma-related hospitalization and ED visits were included if asthma (ICD-9-CM code 493) was assigned in the first diagnostic code. Asthma-related outpatient visits were included if asthma was present in any of the first three diagnostic code fields. Medication data, e.g. oral steroid prescriptions, were not electronically available in the REP data linkage system at the time of this study. For each individual, the mean number of asthma exacerbations experienced per year was calculated by dividing the total number of asthma exacerbations experienced by the individual during 2000–2010 by the number of years the individual was an Olmsted County resident during the same time period. Years of residence in Olmsted County was determined for each year of the study based on inclusion in the REP system.

The case definition for asthma has been tested against medical record review with specificity of over 98 %. Sensitivity is more difficult to obtain but compared to other definitions of asthma case status, the case definition used was over 96 % sensitive [39, 40]. Using only one diagnostic code for asthma the specificity declines to less than 75 % in children while remaining high for adults (92 %). We decided that a single definition across the age spectrum was our best option. We did not test a more stringent definition.

Demographic data on age and gender were obtained from the individual medical record. Block group level poverty rates were obtained from the US Census Bureau American Community Survey 5-Year Summary File 2010. Each individual in the study was assigned a poverty index level based on the block group poverty level of their most recent address in the REP record at the time the data were extracted (2011). The poverty index was calculated as the percentage of households within each census block group with an income-to-poverty ratio less than one (POV100RATE in the Summary File).

### Traffic exposure

Traffic-related exposure indicators were developed for Olmsted County using traffic count data (AADT – Annual Average Daily Traffic) for the sum of light duty and heavy commercial traffic from the Minnesota Department of Transportation (MNDOT) [41]. Three traffic exposure metrics were calculated: 1) Vehicle Kilometers Traveled (VKT) within 250 m of each individual’s residence (VKT_250); 2) VKT within 500 m of each individual’s residence (VKE_500); and 3) Traffic density (AADT per square meter) calculated with a kernel density algorithm.

VKT were calculated for the year 2009 using Geographic Information System Software (ArcGIS, Version 10, Esri, Redlands, CA) by multiplying the length of each roadway segment within buffers (250 and 500 m) around each residential location by the traffic count on the segment. The VKT were summed for each buffer for each residence.

Traffic density calculations were made for year 2005 using the Hawth’s Tools kernel density function within ArcGIS to generate a traffic density (raster) surface with a resolution of 50 m. A Gaussian algorithm was invoked in which traffic influence became insignificant beyond 300 m. The density value in each cell reflects all the traffic on all the roads within the 300 m distance. The effect of a given roadway segment depends on the amount of traffic on the road segment, its distance from the raster cell, and the form of the algorithm. The units of the metric are traffic counts per square meter per day.

The traffic exposure data and REP asthma patient data were geographically linked in order to assign traffic exposure values to each individual. This was accomplished by geocoding the most recent address of the REP asthma patients using the ArcGIS geocoder tool to obtain the spatial coordinates of the residential street address. The geographic location of unmatched addresses was manually determined with Google Earth (Version 6.0, Google, Mountain View, CA). Most of the Olmsted County patients with asthma lived in or near the city of Rochester, the most traffic-dense region of the county. Three traffic exposure measures were assigned to each individual, the traffic density, VKT within a 250 m buffer (VKT_250), and VKT within a 500 m buffer (VKT_500).

### Statistical analysis

As an initial exploration of the relationship between each of traffic measures and asthma exacerbations, the number of exacerbations was tabulated by quintiles of each of the three traffic metrics. Within each quintile (1-Lowest traffic, 5-Highest traffic) the average exacerbations per year was calculated.

Two multivariate regression models were run to evaluate associations between traffic and asthma exacerbations: 1) a logistic regression model using any asthma exacerbations vs. no exacerbations as the dichotomous outcome variable; and 2) a Poisson regression model using the number of asthma exacerbations/person/year as the outcome variable. Gender, age and age^2^ were included in the models, as well as the poverty index. From the model results, odds ratios and rate ratios were calculated for an increase of 10 % in each traffic measure. The interpretation of the odds ratios are the odds of any asthma exacerbation (logistic) or a rate ratio increase in one exacerbation (Poisson) for a 10 % increase in the traffic measure.

All study procedures were reviewed and either exempted or approved by the Institutional Review Boards of the Minnesota Department of Health and the Olmsted Medical Center.

## Results

There were 19,915 Olmsted County residents diagnosed with asthma included in the analysis of the 2000–2010 REP data. Table 1 shows the distributions of gender, age and poverty index among the participants with asthma diagnoses. The population of asthma patients used in these analyses is younger on average (average age = 27.3) with a higher percentage of females (56 %) than in the Olmsted County population as a whole.

**Table 1:** Number and characteristics of asthma patients included in study

Characteristic	Number	Percent
Total Cases	19,915	100
Gender		
Males	8785	44
Females	11,130	56
Age (years)		
0–4	2853	14
5–9	2272	11
10–19	3953	20
20–64	9436	47
≥ 65	1401	7
Poverty Index (%)		
0	1179	6
1–10	14,494	72
11–20	3781	19
20–30	144	1
> 30	396	2

The majority of adults and children diagnosed with asthma (81.4 %) had no exacerbations requiring an ED visit, hospitalization or three healthcare contacts within any two week window during the 11-year period. Among the patients who had any exacerbations in the study period, 3664 had 1–3 exacerbations and 33 had 4 or more exacerbations.

The mean number of asthma exacerbations per person per year had similar distributions within adult and childhood age categories (adult mean = 0.068, children mean = 0.060, t-test *p* value = 0.287). Female patients had slightly higher, but not significantly different, numbers of exacerbations/year (female mean = 0.067, male mean = 0.062, t-test *p* value = 0.148).

**Fig. 1:**
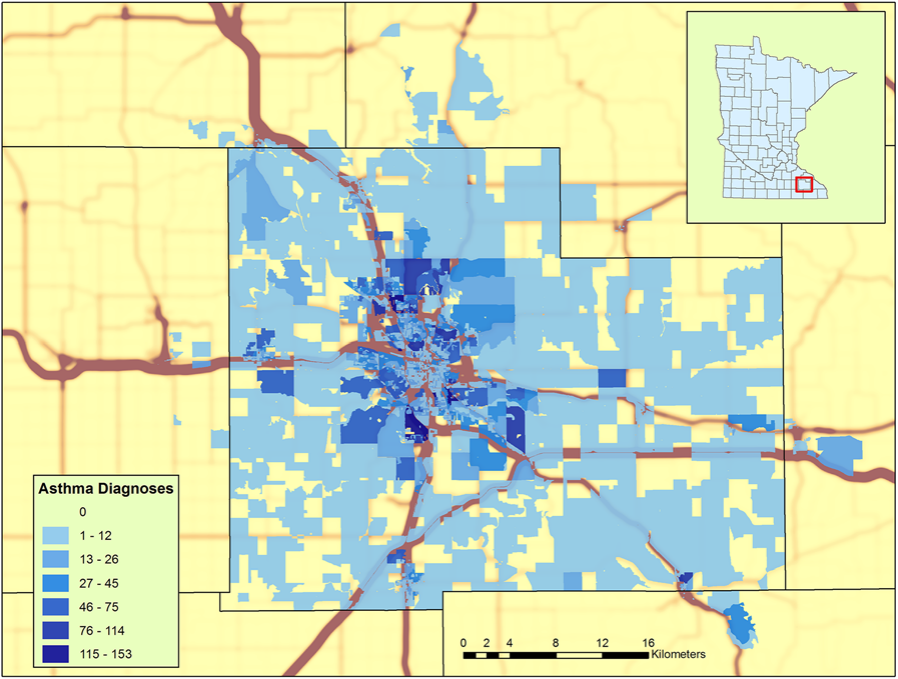
Study location in Olmsted County, Minnesota. The base map is traffic kernel density shading. Census blocks are shaded with increasing intensity according to the number of asthma diagnoses

**Fig. 2:**
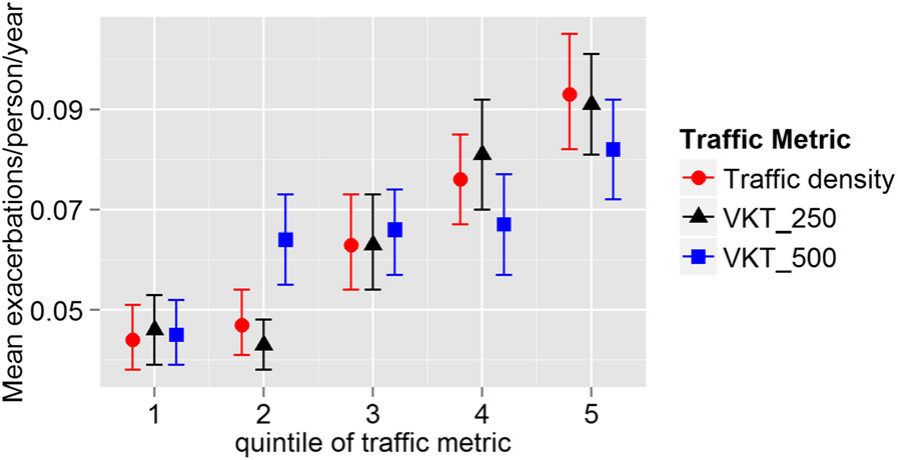
Mean asthma exacerbations per person per year within quintiles of each traffic metric. Traffic density units are traffic counts per square meter per day, and VKT units are vehicle kilometers traveled per day

Figure 1 shows the Olmsted County study area illustrating the traffic density and the number of individuals diagnosed with asthma by census block. Univariate analyses of the association between traffic exposure and asthma exacerbations showed that the mean number of asthma exacerbations experienced per person/year increased with increasing levels of all three traffic exposure measures (traffic density, VKT_250 and VKT_500). Fig. 2 shows the increasing trend of exacerbations with higher levels of traffic exposure. At the highest quintile levels of traffic exposure the mean number of asthma exacerbations per person per year was approximately twice the number in the lowest quintile of traffic exposure.

The multivariate logistic regression models accounted for sex, age and poverty index as well as the three traffic exposure measures. The odds ratios and rate ratios for each model are shown in Table 2. All pairwise interaction terms were entered into the model, but none were significant and they were subsequently omitted. For a 10 % increase in traffic exposure, the odds of experiencing an asthma exacerbation increased as shown in Table 3.

**Table 2:** Results of logistic and Poisson regression analyses of asthma exacerbations and traffic exposure

	Logistic^a^	Poisson^b^
Variable	OR 95 % CL	OR 95 % CL
Age	0.98 (0.97, 0.98)	1.00 (0.99, 1.00)
Sex	1.12 (1.04, 1.20)	1.29 (1.18, 1.29)
Poverty Index	1.02 (1.01, 1.02)	1.02 (1.02, 1.02)
Log Traffic density	1.15 (1.11, 1.20)	1.16 (1.12, 1.16)
Age	0.98 (0.97, 0.98)	1.00 (0.99, 1.00)
Sex	1.12 (1.04, 1.20)	1.30 (1.18, 1.30)
Poverty Index	1.02 (1.01, 1.02)	1.02 (1.02, 1.02)
log VKT 250 m	1.10 (1.08, 1.13)	1.11 (1.08, 1.11)
Age	0.98 (0.97, 0.98)	1.00 (0.99, 1.00)
Sex	1.12 (1.04, 1.20)	1.29 (1.18, 1.29)
Poverty Index	1.02 (1.01, 1.02)	1.02 (1.02, 1.02)
log VKT 500 m	1.06 (1.04, 1.08)	1.08 (1.05, 1.08)

^a^The logistic asthma exacerbation outcome was defined as 1 = any exacerbations/year, 0 = no exacerbations. Sex 1 = male, 0 = female, age, age^2^ and poverty index were modeled as continuous variables

^b^The Poisson asthma exacerbation outcome was defined as number of asthma exacerbations per year. Sex 1 = male, 0 = female, age, age^2^ and poverty index were modeled as continuous variables

**Table 3:** Odds ratios for experiencing an asthma exacerbation given a 10 % increase in each of the three traffic metrics

Effect	Logistic*	Poisson**
	Odds ratio (95 % CI)	Odds ratio (95 % CI)
Traffic Density	1.15 (1.11–1.22)	1.16 (1.12–1.17)
VKT_250 m	1.10 (1.08–1.13)	1.11 (1.08–1.12)
VKT_500 m	1.06 (1.04–1.08)	1.08 (1.05–1.09)

*The logistic asthma exacerbation outcome was defined as 1=any exacerbations/year, 0=no exacerbations

**The Poisson asthma exacerbation outcome was defined as number of asthma exacerbations per year

All models included age (quadratic term), sex and poverty index﻿

The outcome variable in the Poisson multivariate regression models was the mean number of asthma exacerbations experienced per person per year. Traffic metrics were significant predictors of the mean number of asthma exacerbations experienced per person per year in Poisson regression analyses, after accounting for sex, age and poverty index. The regression effect for each model is shown in Table 2. For a 10 % increase in traffic, the odds of experiencing an increase of one asthma exacerbation (on average per year) is shown in Table 3.

In both the logistic regression and Poisson regression analyses, the associations between traffic and asthma exacerbations grew stronger as the defined traffic metric boundaries became more geographically refined. In other words, the odds ratios for the traffic exposure measures were stronger for the 250 m buffer and the traffic density metric than for the 500 m buffer (Table 3).

## Discussion

There are relatively few studies that utilize clusters of asthma clinic visits in addition to hospital and ED visits to measure asthma exacerbations in relation to residential traffic proximity. Our study included visits to outpatient clinics (both those overseen by asthma specialists and primary care physicians), emergency room visits and hospitalizations. Hospitals in the US do not run outpatient specialty clinics, so a hospitalization suggests a moderate to severe asthma episode while less severe ones are addressed in the emergency department or during office hours in outpatient clinics. The combination of the three definitions of exacerbations gives an individual record of the burden of asthma over time across a broad-based population. In addition, the large number of study subjects assigned individual exposure values based on address level data gives considerable power to the analysis.

We found significant relationships between asthma exacerbations and residential proximity to traffic. The probability and number of exacerbations rose with proximity of the residence to traffic. These findings were consistent across all three metrics of traffic exposure and were statistically significant after controlling for gender, age and poverty. Poverty is a well-known risk factor for asthma incidence and exacerbation [9, 15, 16, 20–22]. The number of asthma exacerbations by traffic quintile rose to higher levels and in a more linear fashion for the more spatially resolved metrics, i.e., traffic density and VKT_250, than for the less spatially resolved metric of VKT_500. We infer from this finding that the larger 500 m buffer distance does not capture the traffic effect as well as the more spatially resolved measures. The risk of asthma exacerbations increased in the second quintile of VKT_500 and remained unchanged across a broad range through the third and fourth quintiles suggesting that this metric does not distinguish outcomes except at very high and very low levels. VKT_500 includes traffic from further distances that may not be as important in affecting asthma outcomes. In contrast, the risk of asthma exacerbations remained low over the lowest two quintiles of traffic density and VKT_250, and then increased in a linear fashion through the highest quintile. This finding suggests that low levels of traffic close to the residence may be relatively unimportant, but at higher levels, the risk of asthma exacerbations increases significantly as nearby traffic increases. The mean of the second quintiles of VKT_250 and traffic density were 1420 vehicle kilometers travelled per day and 0.29 traffic counts per square meter per day, respectively. Our results suggest that traffic levels above those values increase the risk of asthma exacerbations.

The odds ratios are stronger for the more spatially resolved traffic metrics (Table 3). These observations are in agreement with studies showing that many traffic-related air pollutants disperse to “background” values over 200–300 m [42–45]. In both logistic and Poisson regressions the strongest traffic metric variable was traffic density, a metric that gives more weight to traffic that is closer to the point of interest, in this case the residence of the asthma patient. We cannot exclude other factors from possible contributions to the distance effect. For example, we know from work in other areas [46, 47] that minorities with higher asthma prevalence may disproportionately reside near high traffic corridors. We did not have access to individual level racial data to explore this issue.

Age was treated as a quadratic variable based on previous work [48]. Our work confirms the curvilinear relationship between asthma exacerbations and age. Adding the quadratic term for age also had the effect of making gender a significant variable in the models. Regression effects for age and gender were nearly identical among all models. We interpret this result as suggesting that the effect of age and gender are not affected by poverty or traffic.

Our use of traffic exposure, rather than exposure to a specific air pollutant (or pollutants) as the predictor of asthma exacerbations has strengths and weaknesses. The main weakness is that the agent or agents in traffic responsible for exacerbating asthma are not identified, and therefore mechanisms are more difficult to elucidate. On the other hand, to the extent that the effect of traffic is attributable to multiple agents acting over the relatively short distances we considered, our method can capture more of the cumulative effects.

Rochester is a relatively small city where traffic levels are lower than in most large metropolitan areas, yet we found a significant relationship between asthma and traffic. The risk of asthma exacerbations continued to increase over the range of traffic exposures in our study area. We speculate that the risk of asthma exacerbations may further increase at the higher levels of traffic exposure found in bigger cities, and we suggest that further study is warranted to investigate this hypothesis.

The city of Rochester and Olmsted County are predicted to grow significantly in coming years. Destination Medical Center is a $5.6 billion economic development project that aims to enhance Rochester’s position as a global health care hub. “The downtown draws 40,000 employees today, but in two decades another 25,000 to 30,000 people could be making that commute. Today about 75 % of all commuters drive to work….” [49]. As documented in this newspaper report, the effects on health and the environment of potential increases in traffic driven by development is a matter of concern in Rochester and elsewhere. Our findings provide information that tends to support those concerns.

Traffic count data were taken as annual average daily traffic values measured in a single year. They do not account for annual, diurnal, day-of-week or seasonal variations in traffic. In addition, the traffic metrics were not matched temporally to the year of a patient record. However, AADT values typically do not vary markedly from year to year except when construction projects and road closures change traffic patterns.

Traffic exposure measures were assigned spatially based on the metric value at the participant’s most recent residential address, as prior residential histories were not available in the REP database. Participants may have moved one or more times during the study period which may result in errors in estimates of traffic exposure. The traffic exposure value does not account for exposures to traffic-related air pollutants that occur away from home, nor does it account for pollutant exposures from indoor sources and outdoor sources other than traffic. Furthermore, we did not examine or control for the effects of individual air pollutants or combinations of pollutants.

Poverty status was not available at the individual or household level, so the block group poverty value was used to represent the poverty status of all individuals in the block group. While block group data may be more homogenous than census tract or zip code data for characterizing poverty status [50], this use of a generalized poverty index value may mis-categorize this variable for many participants. Given the lack of individual or household poverty information, our estimate of the effect of poverty in our population has an uncertainty that we are unable to quantify. Future analyses with other surrogate measures of individual socioeconomic status, such as housing characteristics, are needed [51–53].

## Conclusions

We used a unique medical record data linkage system in Olmsted County, Minnesota to provide a comprehensive picture of asthma exacerbations over 11 years and to develop three address-based metrics of traffic exposure for 19,915 patients. After controlling for demography we found evidence that traffic exposure at the residence increases the risk of asthma exacerbations using all three metrics. These findings support previous studies indicating a relationship between traffic exposure and asthma exacerbation risk. Furthermore, this study demonstrated the application of spatially refined indicators of traffic-related exposure and poverty for monitoring the impact of traffic exposure on asthma patients. Future study could examine refinement of these indicators and monitor how the health of this community may be impacted by anticipated growth of traffic.

## Abbreviations

AADT: Annual average daily traffic
ED: Emergency Department
REP: Rochester epidemiology project
VKT: Vehicle kilometers traveled
VKT_250: Vehicle kilometers traveled within a 250 meter buffer
VKT_500: Vehicle kilometers traveled within a 500 meter buffer
